# On the epicenter of COVID-19 and the origin of the pandemic strain

**DOI:** 10.1093/nsr/nwac286

**Published:** 2022-12-19

**Authors:** Yongsen Ruan, Haijun Wen, Mei Hou, Weiwei Zhai, Shuhua Xu, Xuemei Lu

**Affiliations:** State Key Laboratory of Biocontrol, School of Life Sciences, Sun Yat-sen University, China; State Key Laboratory of Biocontrol, School of Life Sciences, Sun Yat-sen University, China; State Key Laboratory of Biocontrol, School of Life Sciences, Sun Yat-sen University, China; Institute of Zoology, Chinese Academy of Sciences, China; School of Life Sciences, Fudan University, China; State Key Laboratory of Genetic Resources and Evolution; Yunnan Key Laboratory of Biodiversity Information Kunming Institute of Zoology, Chinese Academy of Sciences, China

Ruan *et al*. (2022) published in *National Science Review* (NSR) an analysis that concludes two early centers in the spread of COVID-19 [1]. Since the strain (referred to as DG1111) that spread globally to cause the pandemic has never been found in Wuhan prior to its arrival from outside Asia in March 2020, Wuhan is a local center, rather than the ‘global epicenter’. Citing observations from Europe [2–8], we suggest that the jump from animal hosts to humans must have been earlier than the fall of 2019 to permit the evolution from the ancestral DG0000 strain to the global DG1111 (0 and 1 designating the ancestral and derived variant, respectively).

This essay is written in response to two recent back-to-back publications in *Science* [9,10] that contradict our conclusion. In Worobey *et al*., Wuhan with its seafood market is concluded to be the epicenter of the COVID-19 pandemic. In Pekar *et al*., the appearance of SARS-CoV-2 in humans is estimated to be in November of 2019, presumably in Wuhan. We will first discuss the flaws of the two *Science* publications and expand the scope of our original conclusion as other recent papers have largely corroborated our conclusion.

The flaw in Worobey *et al*. is pointed out by Cao *et al*. Since Worobey *et al*. used only samples from Wuhan to pinpoint the epicenter of COVID-19, they could not possibly have concluded otherwise. The contradiction with our conclusion is hence easily explained.

Similarly, Pekar *et al*.’s interpretation is also conceptually flawed. They estimated tMRCAs of SARS-CoV-2 found in humans, which means the time to the *most* recent common ancestor of the viral variants. However, the authors interpret tMRCA to mean the timing of the viral jump from animal hosts to humans. Imagine that an ancestral SARS-CoV-2 invaded human populations, say, 50 years ago. Since then, the viral population has undergone a series of genetic changes due to genetic drift and selective sweep. The continual losses and gains of genetic diversity may result in the re-building of the extant diversity only three months ago, which is the tMRCA. Therefore, the most recent common ancestor is usually much younger than the time of invasion. Using the logic of Pekar *et al*., we would have concluded that humans separated from chimpanzees about 0.5 M years ago, which is the tMRCA of human genomic variations, instead of the 6 M years commonly accepted.

The two issues we have to discuss are (i) the invasion of the virus such as SARS-CoV-2 from animal hosts to humans; and (ii) the subsequent spread of the virus in human populations. For viral pathogens, the place of origin (referred to as PL0 for Place zero) is usually conflated with the beginning of the spread (PL1, Place of the first epidemic) [11]. The reasons and evidence from prior epidemics for separating PL0 and PL1 have been extensively discussed [11–16]. Generally, PL0 would more likely be the countryside or wildlife preserve where human density is low but, for years, local humans have been in frequent contact with animal hosts. In contrast, PL1 should be a settlement of high human density that facilitates human-to-human transmission after the virus arrived from PL0. PL1 thus corresponds to the general understanding of the ‘epicenter’ of pandemics.

We now consider the arrival of the virus from PL0 to PL1 when it is ready to invade. Distance between sites may not be a determining factor, given that humans and bats (the most likely mammalian hosts for their high-density habitation) are highly mobile. There are, nevertheless, empirical observations that epidemics are highly stochastic events. Using the branching process of probability, Ruan *et al*. (also see Kucharski *et al*. [17]) have shown that invasions into a new population could only sporadically trigger local epidemics in the early phases of an epidemic. Local epidemics may even reach a moderate level of infections before fading out on its own. In this sense, there may be many false PL1’s (that rise and fall) preceding a true PL1 that eventually establishes itself and succeeds in exporting the epidemic far and wide.

The analyses in Ruan *et al*. [1] focus on the very first new strain of SARS-CoV-2 which emerged in late 2019. This new strain bears 4 mutations of the DG group (C241T, C3037T, C14408T and A23403G; the latter one being the D614G amino acid change). The new strain (or haplotype), designated DG1111, replaces the ancestral DG0000 as it has a much higher fitness than DG0000 or each of the intermediate haplotypes. The replacement was rapid and DG1111 quickly became the foundation of the global pandemic by March–April of 2020. Importantly, the haplotype DG1111 did not exist in Asia until March of 2020 and remained far less common in Asia than in Europe for several more weeks [18,19], which indicates that DG1111 originated outside of Asia, likely from Europe. Nevertheless, it may have been believed that DG1111 in Europe must have descended from an immediate ancestor that spread from Wuhan between December 2019 and January 2020. In this belief, Wuhan is still the likely epicenter of the global pandemic.

Importantly, Ruan *et al*. [1] reject the assumption that DG1111 ultimately traces back to Wuhan. By reconstructing the intermediate haplotypes (DG 1000, 0001, … 1110, … 0111, etc.) and tracking their occurrences, Ruan *et al*. ruled out that DG1111 was a descendent of any strain from Wuhan or, indeed, Asia. If the DG1111 strain in Europe, in the most favorable estimation, is indeed derived from the strain (DG0000) in Wuhan or Asia, it would have taken only a week for DG0000 to evolve into DG1111, after its arrival in Europe. A far more plausible scenario supported by documented studies [2–8] is that DG1111 existed in Europe before any strain could have arrived from Wuhan.

In Ruan *et al*.’s conclusion, DG1111 originated in the true PL1 to become the foundation of the pandemic. The documented occurrences of the DG1111 strain in northern Italy in the last few months of 2019 hinted at the possible location of this PL1 [2,3], which is also supported by unexpected detection of SARS-CoV-2 antibodies in the pre-pandemic period [4–6]. Canuti *et al*. (2022) have recently reviewed the evidence that SARS-CoV-2 had been circulating prior to December of 2019 [20]. Wuhan appears to be a false PL1 because strains found in Wuhan did not leave any descendants in the form of DG1111 that spread globally to cause the pandemic.

Finally, the ‘smoking-gun’ evidence for SARS-CoV-2 origin would be the identification of a relative in wild animals that are ‘sufficiently’ close to the first SARS-CoV-2 strain in humans (Strain #1 for short). At present, the closest relatives were discovered in SE Asia (including the southwestern Yunnan province, which is geographically SE Asian). The similarity measures were, respectively, 96.1% (for RaTG13) and 96.8% (for BANAL-52) to Strain #1 [21,22]. Converting the similarity to the proper measure of genetic divergence (dS value), we obtain 0.12 and 0.14 for the two bat-borne strains. That would be >100 years of separation between them and Strain #1. A further extensive survey [16] of bat-borne coronaviruses consists of more than 700 sampling sites and 13 000 bats. This collection yields a number of SARS-CoV-1–like species that are related to the 2003 SARS virus. Importantly, no SARS-CoV-2 related virus was found in the expanded efforts.

That SARS-CoV-2 has emerged ‘anywhere but here’, to borrow the pronouncement from a recent *Science* commentary [23], is a serious scientific conclusion. The hint that the failure to find SARS-CoV-2 related virus in the large samples is merely an extension of government policy is a curious fantasy. Of course, a more accurate phrase would be ‘somewhere, but probably not China’. The conclusion is based on the evolutionary genetics of SARS-CoV-2 in the early phase of COVID-19 and the extensive (but unsuccessful) attempts to find the ‘smoking gun’ in China's wildlife or in the neighboring regions of SE Asia. It is justifiable for Wu *et al*. to suggest more inclusive searches for SARS-CoV-2 related viruses [15,16], where bats are common and epidemiological SARS-CoV-2 data in 2019 are suggestive.
